# Soil microbial responses to long-term agricultural practices under no-till: insights from depth-resolved sampling of shallow soil layers

**DOI:** 10.1093/femsec/fiag108

**Published:** 2026-10-05

**Authors:** Melina Soledad Piacenza, Silvina Bacigaluppo, Mariana Maury, Fernando Salvagiotti, Frank Guzman, Esteban G Jobbágy, Valeria S Faggioli

**Affiliations:** Grupo de Estudios Ambientales, Instituto de Matemática Aplicada San Luis (IMASL), Universidad Nacional de San Luis, Consejo Nacional de Investigaciones Técnicas, Científicas (CONICET), Av. Italia 1556, D5700 San Luis, Argentina; Suelo, Agua y Manejo de Cultivos, Estación Experimental Agropecuaria (EEA) Oliveros, Instituto Nacional de Tecnología Agropecuaria (INTA), Ruta Nacional 11 km 353, C2206 Oliveros, Santa Fe, Argentina; EEA, INTA, Marcos Juárez, Ruta 12 km 36, X2580 Marcos Juárez, Córdoba, Argentina; Suelo, Agua y Manejo de Cultivos, Estación Experimental Agropecuaria (EEA) Oliveros, Instituto Nacional de Tecnología Agropecuaria (INTA), Ruta Nacional 11 km 353, C2206 Oliveros, Santa Fe, Argentina; Laboratorio de Biomoléculas, Universidad Peruana de Ciencias Aplicadas, Av. Alameda San Marcos 11, 15067 Chorrillos, Lima, Perú; Grupo de Estudios Ambientales, Instituto de Matemática Aplicada San Luis (IMASL), Universidad Nacional de San Luis, Consejo Nacional de Investigaciones Técnicas, Científicas (CONICET), Av. Italia 1556, D5700 San Luis, Argentina; EEA, INTA, Marcos Juárez, Ruta 12 km 36, X2580 Marcos Juárez, Córdoba, Argentina; CABANA Netwotark (Capacity Building for Bioinformatics in Latin America), San Pedro de Montes de Oca, 11501 San José, Costa Rica

**Keywords:** soil microorganisms, soybean monoculture, crop rotations, cover crops, chemical stratification, soil heterogeneity

## Abstract

Fungal and bacterial communities were characterized at three soil depths (0–3.5 cm, 3.5–7.5 cm, and 7.5–15 cm) in a 15-year no-till field experiment involving diversified crop rotations with two levels of nitrogen fertilization and soybean monoculture. We hypothesized that community patterns associated with agricultural practices would be most evident in the uppermost soil layer, and that potentially plant-pathogenic taxa would be more abundant under soybean monoculture. Bacterial community composition varied with soil depth but not among agricultural practices. In contrast, fungal community composition was associated with both soil depth and practice. The only significant pairwise difference was found between soybean monoculture and the most diversified rotation involving cover crops and high nitrogen fertilization. When practices were compared within each soil layer, differences in both bacterial and fungal composition were detected only in the uppermost layer. Here, the bacterial community was associated with extractable phosphorus and pH. Few bacterial bioindicator taxa were detected, and no potentially plant-pathogenic taxa were found under soybean monoculture. These findings highlight the importance of considering chemical gradients across soil depths when evaluating microbial responses to long-term no-till agricultural practices, as management effects may not be expressed uniformly throughout the shallow soil layers.

## Teaser

The effects of agricultural practices on fungal and bacterial communities were most evident in the 0–3.5 cm layer, where bacterial composition was associated with chemical properties, highlighting depth-dependent effects under long-term no-till. The graphical abstract has been created with Canva at the canva.com website.

## Introduction

No-till (NT) cropping systems have been widely adopted as an alternative to conventional tillage, incorporating crop rotation and cover crops as key management components (Basche et al. 2016, Simão et al. 2023, Semmartin et al. 2023). Argentina, a dominant global exporter of soybean and maize, concentrates its production in the Pampas region, a vast agricultural ecoregion covering more than 500 000 km² of arable land, where NT has been adopted in around 80% of the area since the early 1990s (Viglizzo et al. 2003, Jobbágy et al. 2021). The combined adoption of low-diversity crop rotations, increased use of agrochemicals to control weeds, and improvements in fertilization strategies has led to a substantial increase in crop yields (Alvarez and Steinbach 2009, Jobbágy et al. 2021, De Abelleyra et al. 2024). Meanwhile, one defining feature of long-term NT soils is the development of strong vertical chemical stratification, driven by the accumulation of organic matter and nutrients in the surface layers and their progressive decline with depth (Franzluebbers 2002, Blanco-Canqui and Lal 2008). This stratification can be further modulated by crop rotation and fertilization, as rotations incorporating cover crops may enhance soil organic carbon (SOC) in topsoil (Alhameid et al. 2017, Semmartin et al. 2023), whereas long-term N fertilization under NT can lead to surface acidification, particularly at high application rates (Souza et al. 2023, Obour et al. 2023, Pin Viso et al. 2024).

Soil hosts highly diverse communities of fungi and bacteria that play essential roles in organic matter decomposition, nutrient cycling, and the maintenance of soil health (Fierer et al. 2007, Bardgett and Van der Putten 2014, Zhan 2024, Das et al. 2025). These microbial communities are highly sensitive to agricultural management. Increasing crop diversity through rotation tends to enhance soil microbial richness relative to monocultures or less diversified rotations (Venter et al. 2016, Liu et al. 2023, Sun et al. 2024). Moreover, rotations incorporating cover crops have also been associated with increased microbial alpha diversity (Schmidt et al. 2019, Kim et al. 2020, Muturi et al. 2024). Changes in resource availability largely mediate such effects, as crop rotations modify the quantity and quality of root exudates and soil nutrients, thereby reshaping bacterial and fungal diversity (Ai et al. 2018, Fang et al. 2024). These management-driven shifts are often reflected in changes in the relative abundance of specific taxa, allowing the identification of bioindicators associated with specific agricultural practices (Jiménez-Bueno et al. 2016, Zong et al. 2024). For instance, crop rotation has been shown to increase the relative abundance of beneficial fungal genera such as *Humicola* and *Schizothecium*, whereas continuous mono-cropping promotes pathogenic taxa including *Gibberella, Dactylonectria*, and *Neocosmospora* (Dou et al. 2025).

In NT systems of the Argentinean Pampas, contrasting crop rotation systems consistently shaped bacterial alpha diversity and community composition (Figuerola et al. 2015, Fernandez-Gnecco et al. 2021, Frene et al. 2022, 2024), with more diversified rotations favoring copiotrophic taxa and potentially beneficial microorganisms (Fernández-Gnecco et al. 2021, Frene et al. 2022). Specific bacterial taxa have also been identified as potential indicators of agricultural management quality in the region, discriminating between sustainable and nonsustainable practices (Figuerola et al. 2012). It has been observed that the composition of bacterial and fungal communities exhibits differential responses to agricultural management, with bacteria responding more rapidly to the presence of roots from different crops, whereas fungi are more closely linked to long-term organic matter accumulation (Fernandez-Gnecco et al. 2021, Gortari et al. 2026). In addition, some agricultural practices may favor potentially pathogenic fungal taxa, which are often more abundant under soybean monoculture (Fernández-Gnecco et al. 2021, Barbero et al. 2025).

Previous works have evaluated bacterial communities across soil depth, but focused on comparing conventional tillage and NT systems in a single rotation (Gabbarini et al. 2021) or on assessing the legacy effects of contrasting nitrogen fertilization regimes (Pin Viso et al. 2024). These studies provided an important basis for understanding bacterial responses to agricultural management in the region. However, they did not evaluate fungal communities or assess the effects of contrasting crop management systems at different soil depths within a long-term field experiment. Consequently, how contrasting agricultural management systems jointly shape fungal and bacterial communities at different soil depths under continuous NT remains poorly understood. Our study addresses this gap by assessing fungal and bacterial alpha diversity and community composition in three soil layers (0–3.5 cm, 3.5–7.5 cm, and 7.5–15 cm) in a 15-year NT experiment in the Argentinean Pampas. This long-term experiment comprises five contrasting agricultural practices that differ in crop rotation systems, cover crop inclusion, and nitrogen fertilization regimes in cereal phases, representing alternative management strategies commonly used in the region. Our experimental design provides an opportunity to evaluate how long-term agricultural management under continuous no-tillage can modulate microbial communities at three depths within the upper 15 cm of soil. We hypothesized that contrasting agricultural practices would be related to differences in soil microbial alpha diversity and community composition, with management-related differences expected to be more evident in the 0–3.5 cm layer than at greater soil depths, where long-term NT is associated with stronger vertical stratification of soil chemical properties (e.g. SOC, extractable P, pH, and EC). We further hypothesized that contrasting long-term management systems would be associated with distinct microbial taxa at each soil depth, with continuous soybean monoculture favoring potentially plant-pathogenic taxa.

## Materials and methods

### Description of the study site and long-term experiment

The study was conducted in a long-term experiment established in 2006 at INTA Oliveros research Station (32° 32′ S, 60° 51′ W), Argentina, on a Typic Argiudoll (USDA Soil Taxonomy) Maciel series which is deep, well-drained, and carbonate-free, with a loamy surface horizon (clay 209 g kg^−1^, silt 708 g kg^−1^, and sand 83 g kg^−1^), a predominance of illite in the clay fraction, a cation exchange capacity of 19.4 cmolc kg^−1^ and pH 6 (1/2.5 soil/water). The climate is temperate and humid, with an average annual temperature of 17.6°C and average annual precipitation of 1042 mm, concentrated mainly in spring and summer. The site belongs to the Pampas phytogeographic region (Cabrera 1976). Originally, the site’s vegetation was primarily natural grasslands, but much of it has been altered by agricultural and livestock activities (Piquer-Rodríguez et al. 2018). Before the establishment of the long-term experiment, the paddock was under continuous agriculture for 50 years.

The long-term experiment was carried out under rainfed conditions in a no-tillage system. Treatments consisted of five cropping sequences combining three cash crops—soybean (S), maize (M), and wheat (W)—with the inclusion of wheat (before soybean) and vetch (before maize) as winter cover crops (CC), and two N fertilization strategies for maize and wheat based on yield goals, hereafter described as low (LNF) or high (HNF) rate. These N rates were calculated based on soil nitrate (N-NO₃) levels at sowing (0–60 cm depth) (Table S1), subtracted from a threshold of available N for expected grain yields above and below 10 000 kg ha⁻¹ in maize (Salvagiotti et al. 2011, Correndo et al. 2021) and for grain yields above and below 5000 kg ha^−1^ in wheat (Salvagiotti et al. 2004, Ferrari et al. 2010). The general nutritional management at sowing was described in Frene et al. (2024). Soybean did not receive N fertilization since it is not a recommended practice for this crop. Then, five cropping and fertilization practices (hereafter referred to as practices) were as follows: (1) soybean monoculture without N fertilization (S-S_NoF); (2) maize–soybean–wheat/soybean with low N input (M-S-W/S_LNF); (3) the same crop rotation with high N input (M-S-W/S_HNF); (4) winter cover crop/maize-winter cover crop/soybean-wheat/soybean with low N input (CC/M-CC/S-W/S_LNF); and (5) the same crop rotation with high N input (CC/M-CC/S-W/S_HNF).

### Soil sampling and chemical properties

In June 2021, after the soybean harvest, soil samples were collected at three depths (0–3.5 cm, 3.5–7.5 cm, and 7.5–15 cm) in each practice replication, yielding a total of 45 samples (5 practices × 3 replicates × 3 depths). For each experimental unit, the three depth-specific samples were considered a single soil profile for subsequent statistical analyses. At the time of sampling, all plots were free of actively growing crops, reducing potential short-term effects associated with crop growth and root activity on soil microbial communities (Hinsu et al. 2021). Each sample consisted of five subsamples collected using an auger (2.5 cm diameter) previously sterilized with 70% alcohol. All samples were sieved (2 mm) and stored in cryotubes at −80°C until DNA extraction. An aliquot of soil was used to determine soil chemical properties including SOC, extractable phosphorus (P), pH, and electrical conductivity (EC). Briefly, SOC was determined by the Walkley–Black method, which involves oxidizing organic matter with potassium dichromate and sulfuric acid, followed by titration of the excess dichromate (Walkley and Black 1934). P was determined using the Bray-1 extraction method, which involves extracting soil with a solution of ammonium fluoride and hydrochloric acid. The extracted phosphorus was then measured colorimetrically (Gutiérrez Boem et al. 2011). Soil pH and EC were measured in a soil:water mixture (1:2.5 w/v) using a compound electrode (Accumet, Westford, MA, USA).

### DNA extraction and sequencing

Total DNA was extracted from 250 mg of soil using a DNeasy® PowerSoil® Pro-Kit (Qiagen, Hilden, Germany), according to the manufacturer’s instructions. DNA integrity was evaluated by agarose gel electrophoresis, while DNA concentration and purity were measured using a NanoDrop 8000 spectrophotometer (Thermo Fisher Scientific, MA, USA). Amplicon library preparation and sequencing were performed at the Unidad de Genómica y Bioinformática (UGB) of the Instituto Nacional de Tecnología Agropecuaria (INTA, Hurlingham, Buenos Aires, Argentina). For prokaryotes, the hypervariable V3–V4 regions of the 16S rRNA gene were amplified using the primers 341F (5′-CCTAYGGGRBGCASCAG-3′) and 806R (5′-GGACTACNNGGGTATCTAAT-3′) (Yu et al. 2005). For fungal communities, the internal transcribed spacer 2 (ITS2) region was amplified using the primers ITS3_KyO2F (5′-GATGAAGAACGYAGYRAA-3′) and ITS4R (5′-TCCTCCGCTTATTGATATGC-3′) (Toju et al. 2012). Equal amounts of each Polymerase Chain Reaction (PCR) amplicon were pooled and purified using Agencourt AMPure XP beads (Beckman Coulter) following the manufacturer’s instructions. The concentration of the pooled library was quantified using a Qubit and fragment size was determined on Fragment Analyzer (Agilent, Santa Clara, CA, USA). The final pooled library was sequenced on Illumina MiSeq with a MiSeq Sequencing Reagent Kit v2 to obtain 250-bp paired-end reads, with a target sequencing depth of 0.1 M reads per sample.

### Bioinformatic analysis

Bacterial and fungal amplicon sequences were processed using QIIME 2 amplicon version 2024.5 (Bolyen et al. 2019). Denoising was performed using DADA2 (Callahan et al. 2016), as implemented in the QIIME 2 pipeline. DADA2 uses a parametric model to infer biological sequences from sequencing reads, remove chimeric and low-quality sequences, and merge paired-end reads. Specifically, reads with a Phred quality score >25 and a minimum fragment length of 220 bp were retained. An amplicon sequence variant (ASV) table was generated using DADA2. Prokaryotic ASVs were taxonomically assigned using the SILVA database version 138 (Quast et al. 2013), whereas fungal ASVs were taxonomically assigned using a classifier trained on the UNITE v8.2 database (Nilsson et al. 2019) with the *q2-classify-sklearn* plugin. Sequences assigned to mitochondria and chloroplasts were removed from downstream analyses. Raw sequencing reads are available in the NCBI Sequence Read Archive under BioProject accession numbers PRJNA1362405 for fungi and PRJNA1362266 for bacteria.

### Statistical analyses

All statistical analyses were performed in RStudio version 4.4.1 (R Core Team 2024) using the microeco package version 1.16.0 (Liu et al. 2021) for microbial community analysis. The significance level was defined at *P* ≤ .05 for all analyses. Soil chemical properties were analysed using generalized linear mixed models (GLMMs) with a Gamma distribution and log link, fitted using the glmmTMB function from the glmmTMB package version 1.1.10 (Brooks et al. 2017). Models included agricultural practice, soil depth, and their interaction as fixed effects, while samples from the same soil profile were included as a random effect to account for the nonindependence of samples (Jiao et al. 2018). When a significant practice × depth interaction was detected, pairwise comparisons among practices were performed within each soil layer. When the interaction was not significant, the most parsimonious model based on Akaike’s Information Criterion (Akaike 1998) was selected to evaluate the main effects of the explanatory variables. The significance of explanatory variables was assessed using Analysis of Deviance with Type III Chi-squared tests for models including the interaction term and Type II Chi-squared tests for models without the interaction. Estimated marginal means were computed using the emmeans package version 2.0.3 (Lenth 2026), and pairwise comparisons were adjusted using Tukey’s method for multiple testing. Model diagnostics were conducted using the DHARMa package version 0.4.7 (Hartig 2020).

Alpha diversity was assessed using observed richness and the Shannon index. Observed richness was analysed using GLMM with a negative binomial distribution to account for overdispersion (Zuur et al. 2009). Shannon index was analysed using a linear mixed model using the lmer function version 1.1–35.5 of the lme4 package (Bates et al. 2015). Both models included agricultural practice, soil depth, and their interaction as fixed effects, with soil profile as a random effect. The same model selection, *post hoc* comparison, and significance-testing procedures described for soil chemical properties were applied to alpha diversity analyses. The relationship between soil chemical properties and alpha diversity was assessed using Spearman’s rank correlation analysis with the cor.test function from the stats package version 4.4.1 (R Core Team 2024). In the correlation plots, the direction of the correlations (rho) is represented by a color gradient, while their strength (i.e. the absolute value of rho) is indicated by the size of the circles.

To visualize the taxonomic composition of fungal and bacterial communities, the relative abundance of each phylum was calculated for each sample and plotted as stacked bar charts, grouped by agricultural practice and soil depth. To evaluate differences in fungal and bacterial community composition among agricultural practices and soil depths, PERMANOVA based on Bray–Curtis dissimilarity was first performed, including practice, depth, and their interaction as factors. To assess whether management-related differences would be more pronounced in the uppermost soil layer, the dataset was subsetted by soil depth, and a PERMANOVA was performed within each soil layer. These analyses were performed with the cal_manova function of the microeco package. Distance-based redundancy analysis (dbRDA) (Legendre and Anderson 1999) was performed using all measured soil chemical properties as constraining variables to explore their relationships with fungal and bacterial community composition. Following the approach used for PERMANOVA, dbRDA was applied both to the global dataset and within each soil layer using the microeco package. The significance of the dbRDA model was assessed using permutation tests implemented in the vegan package v2.7–1 (Oksanen et al. 2025). The association between individual soil variables and the ordination was evaluated using the cal_ordination_envfit function. The significance of the dbRDA axes and the contribution of soil variables to the explained variation were assessed using the cal_ordination_anova function, based on permutation tests. Homogeneity of multivariate dispersions was tested using the betadisper function from the vegan package, followed by Tukey’s HSD test. All permutation-based tests were performed using 9999 permutations.

To identify differentially abundant taxa among agricultural practices, differential abundance analysis (DAA) was performed at the phylum, family, and genus levels using Analysis of Compositions of Microbiomes with Bias Correction 2 (ANCOM-BC2; Nearing et al. 2022, Lin and Peddada 2024), excluding rare taxa with a mean relative abundance below 1% (i.e. <0.01). Similar to the previous analyses, ANCOM-BC2 was conducted on the global dataset and within each soil depth layer, using agricultural practice as the main factor and S-S_NoF as the reference group. A positive log fold change (LFC) indicates a higher relative abundance in the target practice compared to S-S_NoF. Taxa identified as differentially abundant were considered bioindicators of agricultural practice with which they were associated. Adjusted *P*-values were computed using the Holm method. Fungal ASVs were functionally annotated using FUNGuild (Nguyen et al. 2016), and the relative abundance of the plant pathogen guild was analysed using GLMM with beta distribution, with practices as a fixed effect and samples from the same soil profile as a random effect.

## Results

### Sequencing

Fungal diversity analysis yielded 11 751 ASVs. Prokaryotic diversity was studied using high-throughput sequencing, which identified a total of 38 905 ASVs from the soil samples. After taxonomic classification, a total of 321 and 1329 taxonomic units were retained for fungi and bacteria, respectively. Rarefaction curves based on high-throughput sequencing data flattened, indicating that all samples reached the saturation point and that most of the biodiversity present in the samples was captured for fungi (Fig. S1A) and bacteria (Fig. S1B).

### Soil chemical properties across agricultural practices and soil depth

SOC content and pH differed significantly across the interaction between practice and soil depth (Table 1). Specifically, within the 3.5–7.5 cm layer, CC/M-CC/S-W/S_HNF exhibited significantly higher SOC content than all other practices (Table 2). Conversely, in the uppermost layer (0–3.5 cm), S-S_NoF showed the highest pH values (Table 2).

**Table 1:** Analysis of deviance for soil chemical properties as a function of agricultural practice, soil depth, and their interaction. SOC = soil organic carbon; EC = electrical conductivity; and P = extractable phosphorus. Significance codes: * *P* < .05; ** *P* < .01; and *** *P* < .001.

	SOC (g kg^−1^)			pH		
	χ^2^	df	*P*-value	χ^2^	df	*P*-value
Practice	6.59	4	.16	46.14	4	< .001***
Depth	127.15	2	< .001***	9.75	2	.008**
Practice × Depth	18.80	8	.02*	104.47	8	< .001***
	EC (dS m^−1^)			P (mg kg^−1^)		
	χ^2^	df	*P*-value	χ^2^	df	*P*-value
Practice	3.51	4	.048	4.4	4	.35
Depth	88.34	2	< .001***	867.3	2	< .001***
Practice × Depth	6.89	8	.55	11.6	8	.17

**Table 2:** Soil chemical properties across agricultural practices at each soil layer. Values are presented as mean ± standard deviation. Different lowercase letters indicate *post hoc* comparisons (*P* < .05). Crop abbreviations: maize (M), soybean (S), and wheat (W); cover crop (CC). Nitrogen fertilization regimes: low (LNF) and high (HNF). For EC and P, pairwise comparisons among practices were not performed, as significant variation was observed only across depths (Table 3).

Depth (cm)	Practice	SOC (g kg^−1^)	pH	EC (dS m^−1^)	P (mg kg^−1^)
0–3.5	CC/M-CC/S-W/S_HNF	21.8 ± 1.6 a	6 ± 0.2 b	0.1 ± 0	60.3 ± 11.9
	CC/M-CC/S-W/S_LNF	21 ± 2.1 a	6.2 ± 0.2 b	0.1 ± 0	46.3 ± 20.4
	M-S-W/S_HNF	19.2 ± 1.9 a	5.9 ± 0.2 b	0.1 ± 0	42 ± 23.3
	M-S-W/S_LNF	19.6 ± 1.4 a	6.1 ± 0.1 b	0.1 ± 0	36.7 ± 9.5
	S-S_NoF	18.7 ± 1.7 a	6.7 ± 0.3 a	0.1 ± 0	53.7 ± 7.4
3.5–7.5	CC/M-CC/S-W/S_HNF	16.6 ± 5.4 a	5.9 ± 0.2 a	0.1 ± 0	30 ± 3.5
	CC/M-CC/S-W/S_LNF	12.6 ± 0.6 b	6 ± 0.1 a	0.1 ± 0	21.3 ± 10.7
	M-S-W/S_HNF	12.6 ± 0.6 b	5.9 ± 0.2 a	0.1 ± 0	25.7 ± 20.2
	M-S-W/S_LNF	11.7 ± 0.4 b	5.9 ± 0.1 a	0.1 ± 0	19.7 ± 6.1
	S-S_NoF	12.1 ± 0.1 b	6.1 ± 0.3 a	0.1 ± 0	32.3 ± 6.7
7.5–15	CC/M-CC/S-W/S_HNF	11.1 ± 0.6 a	6 ± 0.2 a	0.04 ± 0.01	15.7 ± 2.1
	CC/M-CC/S-W/S_LNF	11 ± 0.7 a	6 ± 0.2 a	0.03 ± 0.01	11.7 ± 5.1
	M-S-W/S_HNF	11.3 ± 1.1 a	6.1 ± 0.2 a	0.03 ± 0.01	16.7 ± 14.2
	M-S-W/S_LNF	10.5 ± 0.5 a	6 ± 0.1 a	0.03 ± 0.01	10.3 ± 4.7
	S-S_NoF	10.4 ± 0.5 a	6.1 ± 0.1 a	0.04 ± 0.01	16 ± 5.3

A general trend of vertical stratification was observed throughout the sampled soil profile (Table 3). SOC content peaked in the uppermost layer (0–3.5 cm) and decreased by ∼35% in the 3.5–7.5 cm layer and ∼46% in the 7.5–15 cm layer. Similarly, pH decreased by 3% in the 3.5–7.5 cm layer, and by 2% in the 7.5–15 cm layer relative to the uppermost layer. EC and extractable phosphorus (P) varied significantly only with depth (Table 1), with both parameters being significantly higher in 0–3.5 cm than in deeper layers (Table 3). Notably, P content dropped sharply by ∼46% from the 0–3.5 cm to the 3.5–7.5 cm layer and by ∼71% from the 0–3.5 cm to the 7.5–15 cm layer.

**Table 3:** Soil chemical properties across depths. Values are presented as mean ± standard deviation. Different lowercase letters indicate *post hoc* comparisons (*P* < .05). For SOC and pH, pairwise comparisons among depths were not performed because the practice × depth interaction was significant; comparisons were therefore performed within each depth (Table 2).

Depth	SOC (g kg ^−1^)	pH	EC (dS m^−1^)	P (mg kg^−1^)
0–3.5	20 ± 1.9	6.2 ± 0.3	0.1 ± 0.01 a	47.8 ± 15.9 a
3.5–7.5	13.1 ± 2.7	6 ± 0.2	0.04 ± 0.01 b	25.8 ± 10.7 b
7.5–15	10.9 ± 0.7	6 ± 0.1	0.03 ± 0.01 c	14.1 ± 6.9 c

### Alpha diversity

Fungal observed richness varied significantly with agricultural practices, soil depth, and their interaction (Table 4). Pairwise comparisons revealed differences among practices at 0–3.5 cm, with M-S-W/S_LNF showing significantly higher richness than S-S_NoF (Fig. 1A). The Shannon index was related significantly with soil depth, with higher values at 0–3.5 cm than in the 3.5–7.5 cm layer; the 7.5–15 cm layer did not differ significantly from either (Fig. 1B). SOC, EC, and P were significantly and positively correlated with both fungal richness and Shannon index, while pH showed a weak but significant correlation only with the Shannon index (Fig. 1C and D).

**Figure 1:**
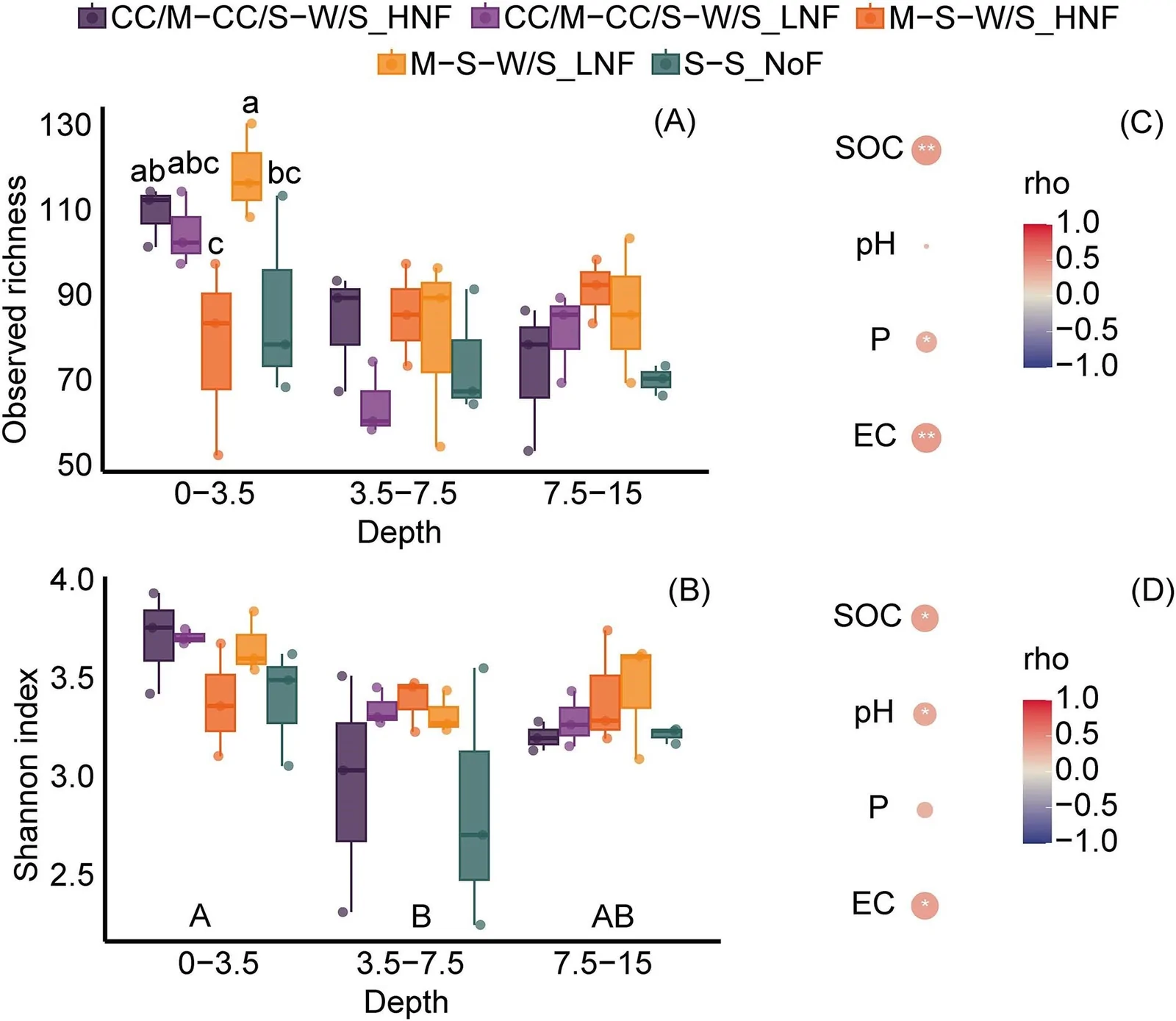
Fungal observed richness (A) and Shannon index (B). Lowercase letters indicate *post hoc* comparisons among practices within each soil depth, and uppercase letters indicate *post hoc* comparisons among depths, based on results in Table 4. Spearman correlations between soil chemical properties and observed richness (C) and Shannon index (D). Circle size represents the magnitude of Spearman’s correlation coefficient (rho), and the gradient represents the direction of the correlation, from positive to negative. Significance codes: * *P* < .05; ** *P* < .01. SOC: soil organic carbon; EC: electrical conductivity; and P: extractable phosphorus.

**Table 4:** Analysis of deviance from mixed-effects models for fungal and bacterial alpha diversity across agricultural practices, soil depths, and their interaction.

	Fungal alpha diversity					
	Observed richness			Shannon index		
	χ^2^	df	*P*-value	χ^2^	df	*P*-value
Practice	18.45	4	.001**	3.92	4	.42
Depth	19.42	2	< .001***	10.29	2	.006 **
Practice × Depth	32.10	8	< .001***	7.22	8	.51
	Bacterial alpha diversity					
	Observed richness			Shannon index		
	χ^2^	df	*P*-value	χ^2^	df	*P*-value
Practice	14.27	4	.006 **	6.88	4	.14
Depth	0.12	2	.94	16.27	2	< .001***
Practice × Depth	26.36	8	.001**	14.89	8	.061

Bacterial observed richness showed significant differences among agricultural practices and a significant practice × soil depth interaction, but not among soil layers (Table 4). Pairwise comparisons detected differences among practices at 0–3.5 cm and 3.5–7.5 cm, but not at 7.5–15 cm (Fig. 2A). Consistent with the findings for fungi, M-S-W/S_LNF exhibited significantly higher richness than S-S_NoF at 0–3.5 cm. The Shannon index was significantly related to soil depth (Table 4), with higher values at 0–3.5 cm than at both deeper layers, which did not differ from each other (Fig. 2B). SOC and EC were significantly and positively correlated with bacterial richness and Shannon index, while pH and P were significantly associated only with Shannon index (Fig. 2C and D).

**Figure 2:**
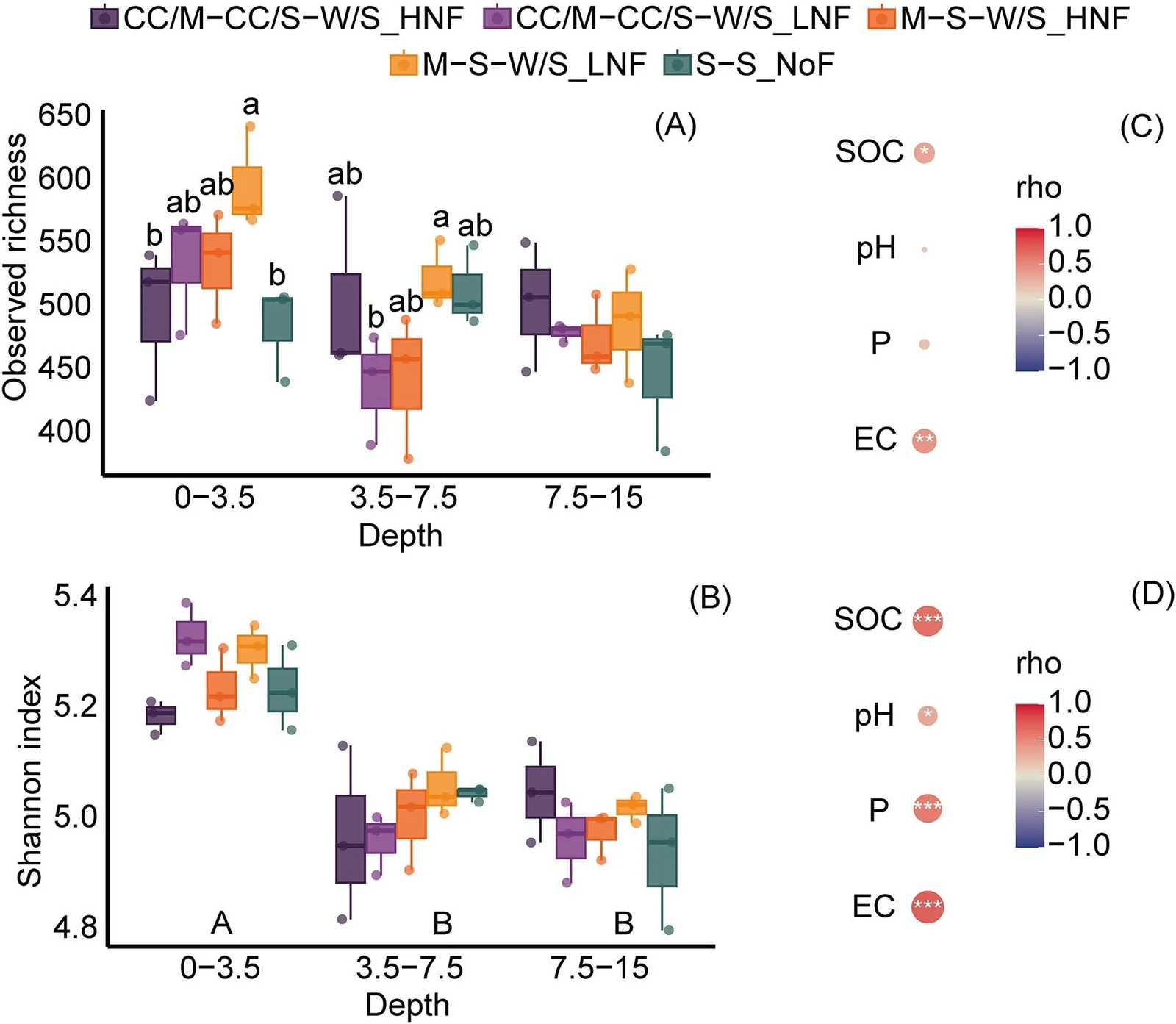
Bacterial observed richness (A) and Shannon index (B). Lowercase letters indicate *post hoc* comparisons among practices within each soil depth, and uppercase letters indicate *post hoc* comparisons among depths, based on results in Table 4. Spearman correlations between soil chemical properties and observed richness (C) and Shannon index (D). Circle size represents the magnitude of Spearman’s correlation coefficient (rho), and the gradient represents the direction of the correlation, from positive to negative. Significance codes: * *P* < .05, ** *P* < .01, *** *P* < .001. SOC: soil organic carbon; EC: electrical conductivity; and P: extractable phosphorus.

### Community composition

Taxonomic classifications were assigned to ASVs based on the closest identifiable taxonomic rank. The phylum *Ascomycota* accounted for ~60% of the fungal relative abundance across all agricultural practices and soil depths. *Rozellomycota* was particularly represented in the CC/M-CC/S-W/S_HNF practice at 3.5–7.5 cm (Fig. S2A). About 75% of bacterial relative abundance across all agricultural practices and soil depths was accounted for by three phyla: *Actinobacteriota, Proteobacteria*, and *Acidobacteria* (Fig. S2B).

Permutational multivariate analysis of variance (PERMANOVA) revealed significant differences in fungal community composition among agricultural practices and soil depths, with no significant practice × depth interaction (Table 5). Pairwise comparisons among practices identified significant compositional differences between S-S_NoF and CC/M-CC/S-W/S_HNF (Table S2). Pairwise comparisons among soil depths indicated that fungal community composition differed significantly across all three of them (Table S3). The dbRDA constrained by all measured soil chemical properties explained 78.4% of the variation in fungal community composition and was statistically significant (Fig. 3A).

**Figure 3:**
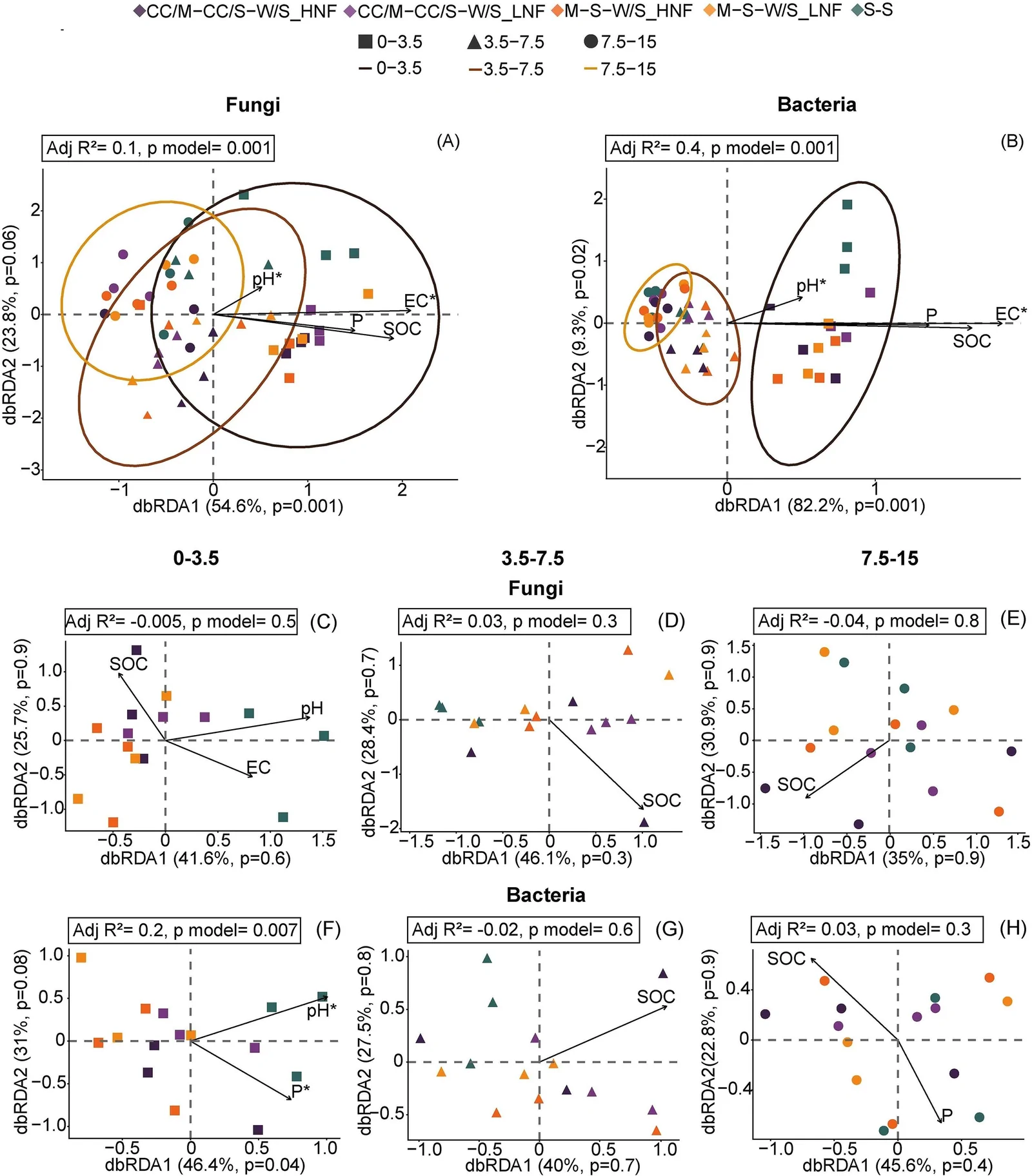
Fungal and bacterial community composition. dbRDA constrained by all measured soil chemical properties for fungal (A) and bacterial (B) communities. Ordinations in A and B include 95% confidence ellipses for each soil depth. Depth-stratified dbRDA are shown separately for fungi at 0–3.5 cm (C), 3.5–7.5 cm (D), and 7.5–15 cm (E), and bacteria at 0–3.5 cm (F), 3.5–7.5 cm (G), and 7.5–15 cm (H). The significance of the dbRDA model and axes was assessed using permutation tests. Vectors represent soil chemical variables significantly correlated with community composition according to envfit analyses, and asterisks indicate variables that were also significant based on permutation tests. Gray lines denote the zero point on both axes.

**Table 5:** PERMANOVA results based on Bray–Curtis dissimilarity for fungal and bacterial community composition in relation to practice, depth, and their interaction (Practice × Depth). *P*-values were adjusted for multiple comparisons using the Bonferroni method. Significance level: *P* = .05. Significance codes: ns (nonsignificant); *** (*P* < .001).

	Fungi		Bacteria	
	*R* ^2^	*P*-value	*R* ^2^	*P*-value
Practice	0.14	.0004 ***	0.07	.12 ns
Depth	0.16	.0001 ***	0.44	.0001 ***
Practice × Depth	0.16	.260 ns	0.12	.20 ns
Residual	0.55		0.37	

To assess whether compositional differences among practices were more pronounced in the uppermost layer, PERMANOVA was conducted separately for each soil layer. Significant differences among practices were detected only in the 0–3.5 cm layer (Table S4); however, no significant pairwise differences were detected within this layer. The dbRDA models were not significant at any depth (Fig. 3C–E). According to envfit analyses, pH, EC, and SOC were significantly correlated with community ordination at 0–3.5 cm (Fig. 3C), whereas only SOC was significant at 3.5–7.5 cm (Fig. 3D) and 7.5–15 cm (Fig. 3E). Permutation tests of the constrained ordination did not identify any significant contribution of soil chemical variables at any depth (*P* > .05; Fig. 3C–E).

For bacterial communities, PERMANOVA detected significant differences in community composition among soil depths, while agricultural practices and the practice × depth interaction were not significant (Table 5). Pairwise comparisons among soil depths indicated that bacterial community composition differed significantly across all three of them (Table S3). The dbRDA constrained by all measured soil chemical properties accounted for 91.5% of the variation in bacterial community composition and was statistically significant (Fig. 3B). Analysis of multivariate dispersion revealed greater within-layer variation in bacterial communities at 0–3.5 cm than at 7.5–15 cm (*P* = 0.02).

Consistent with the pattern observed for fungi, depth-stratified PERMANOVA detected significant compositional differences among practices only at 0–3.5 cm (Table S4), with no individual pairwise comparison reaching significance. The dbRDA for the 0–3.5 cm layer positioned S-S samples in the right quadrants (Fig. 3F), and the overall dbRDA model was significant (Fig. 3F). According to envfit analyses, pH and P were significantly and positively correlated with the ordination at 0–3.5 cm (Fig. 3F), SOC at 3.5–7.5 cm (Fig. 3G), and both P and SOC at 7.5–15 cm (Fig. 3H). Permutation tests indicated that pH and P significantly contributed to the constrained variation at 0–3.5 cm (Fig. 3F).

### Differentially abundant taxa among agricultural practices

DAA using ANCOM-BC2 detected no bioindicator taxa among practices when all soil depths were considered together (*P* > .05), and no potentially plant pathogenic taxa were identified as differentially abundant in S-S_NoF. When practices were compared within each soil layer, no fungal bioindicator taxa were detected (*P* > .05), whereas some differences emerged among bacterial taxa. Bacterial bioindicator taxa were detected at 0–3.5 cm and 3.5–7.5 cm (Table 6). At 0–3.5 cm, *Planctomycetota* and *Solibacteraceae* (*Acidobacteriota*) showed significantly higher relative abundance in M-S-W/S_LNF compared to S-S_NoF (positive LFC), while *Acidothermaceae* (*Acidobacteriota*) and *Acidothermus* (*Acidothermaceae*) showed higher relative abundance in CC/M-CC/S-W/S_HNF than in S-S_NoF. Candidatus *Solibacter* (*Acidobacteriota*) showed significantly higher relative abundance in M-S-W/S_LNF than in S-S_NoF at 0–3.5 cm, but significantly lower relative abundance in CC/M-CC/S-W/S_LNF than in S-S_NoF at 3.5–7.5 cm (negative LFC).

**Table 6:** Differentially abundant bacterial taxa identified by ANCOM-BC2 across agricultural practices within each soil depth. LFC values represent differences relative to S-S_NoF (reference group). Positive LFC indicates higher relative abundance compared to the reference, whereas negative LFC indicates lower relative abundance. SE: standard error. Adjusted *P*-values were computed using the Holm method.

Depth	Taxa	Practice	LFC	SE	*P*-value
0–3.5	Planctomycetota	M-S-W/S_LNF	0.70	0.18	.03
	Solibacteraceae	M-S-W/S_LNF	0.43	0.10	.04
	Acidothermaceae	CC/M-CC/S-W/S_HNF	0.75	0.16	.02
	Acidothermus	CC/M-CC/S-W/S_HNF	0.83	0.15	.01
	Candidatus *Solibacter*	M-S-W/S_LNF	0.48	0.11	.04
3.7–7.5	Candidatus *Solibacter*	CC/M-CC/S-W/S_LNF	-0.23	0.05	.03

## Discussion

Previous studies from the Pampas region under NT systems have shown that microbial community structure can respond to the degree of crop diversification within rotation systems (Frene et al. 2022), seasonality (Fernandez-Gnecco et al. 2021), time under crop cover, and crop phase (Frene et al. 2024). Studies that explicitly examined microbial communities across soil depths have also provided important insights into the vertical organization of soil microbial communities in agricultural systems (Gabbarini et al. 2021, Pin Viso et al. 2024). The present study extends previous research by evaluating contrasting agricultural practices across a vertical soil gradient in the upper 15 cm of the soil profile in a long-term field experiment, while jointly characterizing fungal and bacterial communities.

Soil microbial communities exhibited contrasting patterns associated with agricultural practices, consistent with evidence that bacterial and fungal communities often respond differently to crop rotation systems (Li et al. 2025). We found that fungal communities appeared to be more associated with management practices and soil depth, with significant differences detected between soybean monoculture and the practice with the greatest contrast in crop diversification and N fertilization regimes. Conversely, bacteria were more strongly associated with vertical gradients in soil chemical properties. This differential response may reflect the closer relationship between plants and fungi relative to bacteria, as some fungi form biotrophic interactions with plants and depend more directly on plant-derived resources such as litter and root exudates (Gan et al. 2022). In diversified rotations, each crop species provides distinct inputs of residues and root exudates, potentially increasing the diversity of available resources (Castle et al. 2021, Lu et al. 2024, Li et al. 2025). In addition, the inclusion of cover crops extends the period of active root presence relative to soybean monoculture. Together, crop diversification and the temporal continuity of living roots may have contributed to the progressive structuring of fungal communities and their compositional divergence from the monoculture practice.

### Microbial community structure across practices at each soil layer

Long-term field experiments provide a valuable framework for assessing the cumulative effects of agricultural practices, as management histories and their duration are well documented and can therefore be linked to long-term changes in soil properties (Salvagiotti et al. 2017). In our 15-year NT experiment, we observed a pronounced vertical gradient in soil chemical properties across depths, consistent with previous studies (Franzluebbers 2002, Blanco-Canqui and Lal 2008). In addition, chemical properties varied among agricultural practices, exhibiting distinct depth-specific responses. For instance, the highest pH was observed in the uppermost layer under continuous soybean, likely due to the absence of nitrogen fertilization and the low frequency of roots in the soil, which are well-known drivers of soil acidification (Hinsinger et al. 2003, Souza et al. 2023, Obour et al. 2023, Pin Viso et al. 2024). In contrast, SOC was significantly higher in the subsurface layer under the practice combining a diversified crop rotation, cover crops, and high nitrogen fertilization. This management system has been associated with shifts in SOC content (Zheng et al. 2023), potentially reflecting greater inputs of plant residues and distinct root exudates associated with more diverse cropping systems (Novelli et al. 2017, Lu et al. 2024).

By sampling three soil depths, we found that differences in microbial richness and community composition among agricultural practices were detected primarily in the uppermost layer, where P and EC values were highest. At this depth, bacterial community composition was strongly correlated with P and pH. These associations may reflect the influence of nutrient availability on microbial community structure, as nutrient accumulation in specific soil layers may favor taxa with the corresponding metabolic capabilities (Li et al. 2017). Supporting this interpretation, previous studies in agricultural systems have reported shifts in bacterial community composition associated with P availability (Ran et al. 2021, Tian et al. 2022). Consistent with our findings, soil bacterial community composition has also been associated with soil pH (Fierer and Jackson 2006, Hartman et al. 2008, Jenkins et al. 2009, Lauber et al. 2009, Rousk et al. 2010, Muneer et al. 2022). Notably, in our study, these associations occurred within a relatively narrow pH range (5.9–6.7), suggesting that variation in soil bacterial community composition may be associated with relatively small differences in soil pH, even under moderately acidic to near-neutral conditions. For fungal community composition, the association with SOC within each soil depth was relatively weak, contrasting with previous studies that reported stronger relationships between fungal communities and SOC (Fernández-Gnecco et al. 2021, Gortari et al. 2026). This suggests that, in addition to SOC, other environmental and management-related factors may account for the observed variation in fungal community composition in the uppermost soil layer. These may include unmeasured soil properties, such as moisture, temperature, and nutrient availability, as well as differences in current herbicide regimes between more diverse rotations and soybean monoculture (Druille et al. 2013, Li et al. 2020, Vázquez et al. 2021, Kracmarova et al. 2022). Finally, the Shannon index for both fungi and bacteria did not differ significantly among management practices at any soil layer and was primarily associated with the chemical gradient associated with soil depth. This pattern may indicate that management affected the presence or detectability of less abundant taxa, as reflected by observed richness, without substantially altering community evenness, as captured by the Shannon index (Hill 1973). Overall, these findings partially support our expectation that management effects may be more detectable in the uppermost soil layer.

Although bioindicator taxa were identified, no potentially plant-pathogenic taxa were detected under soybean monoculture within our 15-year NT experiment. *Planctomycetota*, a ubiquitous phylum among the bioindicators in the uppermost layer, plays key roles in global carbon and nitrogen cycling (Wiegand et al. 2018), encompassing a continuum of life strategies ranging from oligotrophic to copiotrophic (Ho et al. 2017, Stone et al. 2023). This pattern is consistent with Frene et al. (2024), who found that the maize crop phase increased the abundance of *Planctomycetes* and enriched several taxa within this phylum, including *Gemmata, Singulisphaera*, and *Pirellula*. Furthermore, *Acidothermaceae* and *Acidothermus*, both belonging to the phylum *Acidobacteriota*, have been reported in association with acidic soils (Sait et al. 2006, Ward et al. 2009). Although the soils in our study were not strongly acidic, these taxa were more prevalent under the agricultural practice with the lowest soil pH, suggesting that relatively small differences in soil acidity may influence their distribution. Similarly, Candidatus *Solibacter* has been linked to an oligotrophic life strategy (Stone et al. 2023), and has been identified as an indicator of low-pH soils and negatively correlated with soil pH in agricultural and other land-use systems (Seuradge et al. 2017).

### Study considerations and future perspectives

Some considerations are worth noting when interpreting our results. First, since samples were collected at a single time point—after the soybean harvest—our results provide a snapshot of microbial community responses to long-term agricultural management. Given the temporal dynamics of soil microbial communities (Fernandez-Gnecco et al. 2021), sampling across seasons could provide a more comprehensive assessment of how these communities respond to agricultural practices. Second, because the study was conducted at a single long-term experimental site, broader landscape-scale variability was not assessed, and studies across multiple locations would help further evaluate the generality of our findings. Finally, the absence of differentially abundant, potentially plant-pathogenic taxa in soybean monoculture should not be interpreted as evidence that management did not affect plant disease risk. Targeted approaches, including disease assessments and taxon- or strain-specific molecular assays, are therefore required to further evaluate the occurrence, abundance, and potential pathogenicity of candidate taxa (Okubara et al. 2005, Sudermann et al. 2024).

## Conclusions

This study assessed fungal and bacterial alpha diversity and community composition in a 15-year NT field experiment, incorporating vertically stratified sampling across contrasting practices, including diversified rotations with two nitrogen fertilization regimes and soybean monoculture. Our results revealed contrasting patterns in fungal and bacterial communities. Fungal community composition differed with both agricultural practices and soil depth, whereas bacterial community composition was primarily associated with chemical gradients across soil depth. Within each soil layer, differences among agricultural practices were most evident in the uppermost layer, where bacterial community composition was associated with extractable phosphorus and pH. A limited number of bacterial bioindicator taxa were associated with specific agricultural practices, but no potentially plant-pathogenic taxa were differentially abundant under soybean monoculture. Our findings indicate that long-term agricultural practices do not affect soil microbial communities uniformly across the upper 15 cm of soil. Consequently, sampling multiple soil depths is important for identifying and interpreting microbial responses to agricultural management, as analyses restricted to a single soil layer may overlook important patterns.

## Supplementary Material

fiag108_Supplemental_Files
