# Supplementary material for: Soil microbial responses to long-term agricultural practices under no-till: insights from depth-resolved sampling of shallow soil layers
Source: FEMS Microbiol Ecol. 2026 Oct 5;102(10):fiag108. doi: 10.1093/femsec/fiag108 (PMC13653337; doi:10.1093/femsec/fiag108)
Supplement: Supplementary file 1 [file fiag108_supplemental_files.zip › Figure S1_Supplementary data_Piacenza et al.docx]

Supplementary data_Figure S1

Figure S1. Rarefaction curves for fungi (a) and bacteria (b). Rarefaction curves based on high-throughput sequencing after taxonomic classification. Colors indicate the practice and depth associated with each sample. All 45 samples are displayed.
