# Supplementary material for: Soil microbial responses to long-term agricultural practices under no-till: insights from depth-resolved sampling of shallow soil layers
Source: FEMS Microbiol Ecol. 2026 Oct 5;102(10):fiag108. doi: 10.1093/femsec/fiag108 (PMC13653337; doi:10.1093/femsec/fiag108)
Supplement: Supplementary file 1 [file fiag108_supplemental_files.zip › Figure S2_Supplementary material_Piacenza et al.docx]

Supplementary data_Figure S2

Figure S2. Relative taxonomic composition of fungal (A) and bacterial (B) communities at the phylum level across practices and depths. Bars represent the mean relative abundance across samples within each group. “Others” (in gray) represents fungal taxa that could not be classified at the phylum level and bacterial phyla with a relative abundance <1%.
