# Supplementary material for: Soil microbial responses to long-term agricultural practices under no-till: insights from depth-resolved sampling of shallow soil layers
Source: FEMS Microbiol Ecol. 2026 Oct 5;102(10):fiag108. doi: 10.1093/femsec/fiag108 (PMC13653337; doi:10.1093/femsec/fiag108)
Supplement: Supplementary file 1 [file fiag108_supplemental_files.zip › Table S1_Supplementary data_Piacenza et al.docx]

Supplementary data_Table S1

Table S1. Average nitrogen (N) application rates (kg ha⁻¹ year⁻¹) for wheat and maize at each practice. Crop abbreviations: maize (M), soybean (S), and wheat (W); cover crop (CC). Nitrogen fertilization regimes: low (LNF) and high (HNF).

| Practice | Crop | Average N application rate (kg ha⁻¹ year⁻¹) |
| --- | --- | --- |
| CC/M-CC/S-W/S_HNF | Wheat | 85 |
| CC/M-CC/S-W/S_HNF | Maize | 92 |
| CC/M-CC/S-W/S_LNF | Wheat | 50 |
| CC/M-CC/S-W/S_LNF | Maize | 73 |
| M-S-W/S_HNF | Wheat | 85 |
| M-S-W/S_HNF | Maize | 88 |
| M-S-W/S_LNF | Wheat | 50 |
| M-S-W/S_LNF | Maize | 68 |
