# Supplementary material for: Soil microbial responses to long-term agricultural practices under no-till: insights from depth-resolved sampling of shallow soil layers
Source: FEMS Microbiol Ecol. 2026 Oct 5;102(10):fiag108. doi: 10.1093/femsec/fiag108 (PMC13653337; doi:10.1093/femsec/fiag108)
Supplement: Supplementary file 1 [file fiag108_supplemental_files.zip › Table S2_Supplementary data_Piacenza et al.docx]

Supplementary data_Table S2

Table S2. Pairwise PERMANOVA results based on Bray–Curtis dissimilarities for fungal community composition among agricultural practices, corresponding to the factor that showed a significant effect in Table 5. P-values were adjusted using the Bonferroni method. Significance codes: ns (non-significant); * (p < 0.05).

|  | Fungi |  |  |
| --- | --- | --- | --- |
| Practices | R^2^ | p-value |  |
| CC/M-CC/S-W/S_HNF vs CC/M-CC/S-W/S_LNF | 0.06 | 1 | ns |
| M-S-W/S_LNF vs CC/M-CC/S-W/S_LNF | 0.10 | 0.68 | ns |
| M-S-W/S_LNF vs CC/M-CC/S-W/S_HNF | 0.08 | 1 | ns |
| M-S-W/S_HNF vs CC/M-CC/S-W/S_LNF | 0.07 | 1 | ns |
| M-S-W/S_HNF vs CC/M-CC/S-W/S_HNF | 0.08 | 1 | ns |
| M-S-W/S_HNF vs M-S-W/S_LNF | 0.05 | 1 | ns |
| S-S_NoF vs CC/M-CC/S-W/S_LNF | 0.13 | 0.08 | ns |
| S-S_NoF vs CC/M-CC/S-W/S_HNF | 0.15 | 0.001 | * |
| S-S_NoF vs M-S-W/S_LNF | 0.09 | 0.90 | ns |
| S-S_NoF vs M-S-W/S_HNF | 0.10 | 0.36 | ns |
