# Supplementary material for: Soil microbial responses to long-term agricultural practices under no-till: insights from depth-resolved sampling of shallow soil layers
Source: FEMS Microbiol Ecol. 2026 Oct 5;102(10):fiag108. doi: 10.1093/femsec/fiag108 (PMC13653337; doi:10.1093/femsec/fiag108)
Supplement: Supplementary file 1 [file fiag108_supplemental_files.zip › Table S3_Supplementary data_Piacenza et al.docx]

Supplementary data_Table S3

Table S3. Pairwise PERMANOVA results based on Bray–Curtis dissimilarities for fungal and bacterial community composition among soil depths, corresponding to the factor that showed a significant effect in Table 5. P-values were adjusted using the Bonferroni method. Significance codes: ** (p < 0.01); *** (p < 0.001).

|  | Fungi |  |  | Bacteria |  |  |
| --- | --- | --- | --- | --- | --- | --- |
| Comparison | R^2^ | p-value |  | R^2^ | p-value |  |
| 0 – 3.5 vs 3.5 – 7.5 | 0.11 | 0.0003 | *** | 0.35 | 0.0003 | *** |
| 0 – 3.5 vs 7.5 – 15 | 0.17 | 0.0003 | *** | 0.50 | 0.0003 | *** |
| 3.5 – 7.5 vs 7.5 - 15 | 0.09 | 0.0027 | ** | 0.14 | 0.0018 | ** |
