# Supplementary material for: Soil microbial responses to long-term agricultural practices under no-till: insights from depth-resolved sampling of shallow soil layers
Source: FEMS Microbiol Ecol. 2026 Oct 5;102(10):fiag108. doi: 10.1093/femsec/fiag108 (PMC13653337; doi:10.1093/femsec/fiag108)
Supplement: Supplementary file 1 [file fiag108_supplemental_files.zip › Table S4_Supplementary data_Piacenza et al.docx]

Supplementary data_Table S4

Table S4. PERMANOVA results based on Bray–Curtis dissimilarity for fungal and bacterial community composition in relation to practice at each soil layer. P-values were adjusted for multiple comparisons using the Bonferroni method. Significance level: p = 0.05. Significance codes: * (p < 0.05).

|  |  | Fungi |  | Bacteria |  |
| --- | --- | --- | --- | --- | --- |
|  |  | R^2^ | p-value | R^2^ | p-value |
| 0-3.5 | Practice | 0.35 | 0.03 * | 0.4 | 0.03 * |
|  | Residual | 0.65 |  | 0.6 |  |
| 3.5-7.5 | Practice | 0.36 | 0.07 | 0.34 | 0.18 |
|  | Residual | 0.64 |  | 0.65 |  |
| 7.5-15 | Practice | 0.34 | 0.06 | 0.23 | 0.88 |
|  | Residual | 0.66 |  | 0.77 |  |
